# A Serological Neoepitope Biomarker of Neutrophil Elastase-Degraded Calprotectin, Associated with Neutrophil Activity, Identifies Idiopathic Pulmonary Fibrosis and Chronic Obstructive Pulmonary Disease More Effectively Than Total Calprotectin

**DOI:** 10.3390/jcm12247589

**Published:** 2023-12-08

**Authors:** Annika Hummersgaard Hansen, Joachim Høg Mortensen, Sarah Rank Rønnow, Morten Asser Karsdal, Diana Julie Leeming, Jannie Marie Bülow Sand

**Affiliations:** Nordic Bioscience, 2730 Herlev, Denmarkjsa@nordicbio.com (J.M.B.S.)

**Keywords:** biomarker, neoepitope, neutrophil activity, IPF, COPD, calprotectin

## Abstract

Neutrophil activation can release neutrophil extracellular traps (NETs) in acute inflammation. NETs result in the release of human neutrophil elastase (HNE) and calprotectin, where the former can degrade the latter and generate protein fragments associated with neutrophil activity. We investigated this in chronic obstructive pulmonary disease (COPD) and idiopathic pulmonary fibrosis (IPF) using the novel neoepitope biomarker CPa9-HNE, quantifying a specific HNE-mediated fragment of calprotectin in serum. CPa9-HNE was compared to total calprotectin. Initially, CPa9-HNE was measured in healthy (*n =* 39), COPD (*n =* 67), and IPF (*n =* 16) serum using a neoepitope-specific competitive enzyme-linked immunosorbent assay. Then, a head-to-head comparison of CPa9-HNE and total calprotectin, a non-neoepitope, was conducted in healthy (*n =* 19), COPD (*n =* 25), and IPF (*n =* 19) participants. CPa9-HNE levels were significantly increased in COPD (*p <* 0.0001) and IPF subjects (*p* = 0.0001) when compared to healthy participants. Additionally, CPa9-HNE distinguished IPF (*p <* 0.0001) and COPD (*p <* 0.0001) from healthy participants more effectively than total calprotectin for IPF (*p* = 0.0051) and COPD (*p* = 0.0069). Here, CPa9-HNE also distinguished IPF from COPD (*p* = 0.045) participants, which was not observed for total calprotectin (*p* = 0.98). Neutrophil activity was significantly higher, as assessed via serum CPa9-HNE, for COPD and IPF compared to healthy participants. Additionally, CPa9-HNE exceeded the ability of non-neoepitope calprotectin serum measurements to separate healthy from lung disease and even COPD from IPF participants, indicating that neutrophil activity is essential for both COPD and IPF.

## 1. Introduction

Chronic obstructive pulmonary disease (COPD) affects millions globally and is a disease driven by inflammation where events of acute exacerbations significantly worsen respiratory symptoms [1,2]. The pathological manifestations and individual disease course are highly heterogeneous [2,3], for which classification systems have been developed to better stratify disease management [4]. Idiopathic pulmonary fibrosis (IPF) is an interstitial lung disease characterized by progressive pulmonary fibrosis and a severe disruption of the balance in extracellular matrix (ECM) remodeling but also inflammatory events that include increased neutrophil recruitment and activation [5]. IPF is a fast-progressing disease with a median survival of 3–5 years from the time of diagnosis [6,7]. Due to the heterogeneity between patients within both IPF and COPD, the disease progression is hard to predict. However, an early diagnosis could benefit patients due to earlier treatment initiation, potentially slowing disease progression. Additionally, the identification of disease phenotypes may enable personalized healthcare. Thus, new biomarkers with a diagnostic and/or a prognostic value are of interest for both COPD and IPF [2,6].

Neutrophils are a highly abundant immune cell type in human blood, act as first responders to tissue injury, and have a high turnover during pathological conditions [8]. Neutrophils have a rapid decay in blood when compared to other leukocytes; therefore, it is a requirement that assessment needs to be performed within hours after blood collection and their isolation, which may prove difficult in practice [9,10,11]. As part of the acute immune response, they are swiftly recruited to the site of injury, where they may interact with cellular and ECM components [12]. When the neutrophil is activated, neutrophil extracellular traps (NETs) are released via vital or suicidal NETosis as a response to inflammation induced by pathogens or other stimuli [13,14]. In vital NETosis, neutrophils can release secretory vesicles in the form of NETs. As part of suicidal NETosis, the neutrophil will undergo cell lysis and cell death to release NETs. Calprotectin is a heterodimeric protein mainly produced by neutrophils but also expressed by other immune cells such as macrophages and monocytes [15]. Calprotectin constitutes up to 60% of neutrophilic cytosolic protein content and is released in abundance as part of a NET response; however, passive calprotectin release from neutrophils has also been shown [15,16]. Furthermore, human neutrophil elastase (HNE) is released during a NET event and cleaves the otherwise proteolytically resistant calprotectin [17,18]. Calprotectin has various extracellular biological purposes, the most extensively studied function being its properties as a potent antimicrobial agent, which inhibits pathogen growth by scavenging and withholding transition metals [19,20]. Studies have indicated that released calprotectin can also act as a chemotactic trigger for immune cells and promote cell adhesion [21]. Additionally, subunits and complexes of calprotectin have been shown to stimulate the expression of pro-inflammatory cytokines at varying degrees, ultimately promoting an inflammatory state further [22,23,24,25]. Apart from inflammation-related responses, calprotectin has been suggested as an inducer of cell apoptosis [26,27,28,29]. Interestingly, previous findings have also indicated that the S100A9 subunit can promote fibroblast proliferation in vitro [30].

An increase in NETs in the sputum has previously been observed for COPD during acute exacerbations, where this increase also correlated to a disease in severity as determined by the Global Initiative for Obstructive Lung Disease (GOLD) classification system [31,32]. Furthermore, an increase in neutrophil activity and intact calprotectin has been associated with IPF phenotypes [5,33]. Previously, calprotectin has been suggested as a therapeutic target for IPF treatment, albeit inflammation and the role of immunity remain a debated subject regarding IPF pathology [34,35]. Immune suppressant treatment has been indicated to have a harmful effect, suggesting that the interplay of the immune system and fibrosis is complex, and the exact immune modulation approach may matter [36,37]. New tools are required to enable assessments that reflect the neutrophil activity or NETosis. Recently, the formation of a specific fragment of calprotectin generated by HNE, the neoepitope CPa9-HNE, has been described [16]. This neoepitope has been shown to be generated by activated, but not inactive, neutrophils in vitro. Furthermore, the stability of the neoepitope was demonstrated in stored blood samples, whereas calprotectin and neutrophils are known to have a short half-life in blood [38,39]. Thus, CPa9-HNE represents a novel and robust method of assessing neutrophil activity in blood by assessing the neoepitope of a specific degradation fragment. We hypothesized that CPa9-HNE could be a novel tool to further elucidate disease mechanisms related to neutrophil activity in COPD and IPF by a simple measurement of the generated neoepitope in stored serum samples since CPa9-HNE reflects the secretion and the degradation of calprotectin that is generated by activated neutrophils. Therefore, we investigated neutrophil activity in the serum of healthy, COPD, and IPF subjects by quantifying CPa9-HNE. Additionally, a head-to-head comparison of this newly developed CPa9-HNE assay and a commercial assay for a total calprotectin in serum was conducted to investigate if there was a difference in diagnostic power between a non-neoepitope and a neoepitope-specific calprotectin assay in IPF and COPD.

## 2. Materials and Methods

### 2.1. Disease Cohorts

CPa9-HNE levels were assessed in serum samples from 39 healthy controls, 16 IPF participants, and 67 clinically stable COPD participants. Additionally, serum samples were collected four weeks later at a follow-up visit for 25 of the COPD participants who were clinically stable. For the second part of this study that compared CPa9-HNE with total calprotectin in the serum, 19 healthy controls, 19 IPF participants, and the 25 COPD participants that participated in the follow-up visit were evaluated using each assay head-to-head. Serum samples were stored at −80 °C until biomarker assessments.

After informed consent and approval by the appropriate Institutional Review Board/Independent Ethical Committee were obtained, the serum was collected from participants and healthy controls. Healthy and IPF subject samples were obtained from the commercial vendors BioIVT (West Sussex, UK) and Discovery Life Science (Los Osos, CA, USA), where available basic demographics are shown in Table 1. Serum samples from participants with COPD were collected for an observational study as described previously [40]. The study complies with the Declaration of Helsinki and Good Clinical Practice Guidelines; moreover, it has been approved by the local ethics committee (protocol number H-6-2013-014) [40]. All participants provided written informed consent before the performance of all study-related assessments. Inclusion criteria were a diagnosis of COPD made by a senior physician and forced expiratory volume during the first second (FEV_1_) was <80% of the predicted value. The exclusion criterion was an acute exacerbation of COPD leading to hospitalization within four weeks prior to blood sampling.

### 2.2. Biomarker Assessments

Calprotectin degradation mediated via HNE was quantified by targeting a specific degradation fragment in a stored serum using the robust, competitive enzyme-linked immunosorbent assay (ELISA) CPa9-HNE, as described previously (cat. no. 1031AE01, Nordic Bioscience, Herlev, Denmark) [16]. Briefly, CPa9-HNE quantifies a highly specific neoepitope of calprotectin in the S100A9 subunit generated via HNE. Biomarker values outside the quantification range were assigned either the lower or upper limit of quantification, as appropriate. Total calprotectin (S100A8/S100A9) measurements in the serum were conducted with a commercially available sandwich ELISA (cat. no. EK-MRP8/14, Bühlmann Laboratories, Schönenbuch, Switzerland) that did not target a neoepitope.

### 2.3. Statistical Analysis

Basic demographics were compared between groups using the Kruskal–Wallis χ^2^ test and two-tailed Mann–Whitney *t*-test, as appropriate. Indications of confounding effects were investigated with respect to age, sex, and body mass index (BMI) when available. A statistically significant difference between the disease groups and the healthy group was determined using the Kruskal–Wallis test with Dunn’s multiple comparisons test. A receiver-operating characteristics (ROC) curve analysis was used to determine the specificity and sensitivity of the disease groups compared to the healthy controls for the serum CPa9-HNE. FEV_1_% predicted and forced vital capacity (FVC) % predicted were evaluated for their correlation to CPa9-HNE levels using Spearman’s correlation test. Furthermore, for the 25 COPD subjects that had participated in a follow-up visit, CPa9-HNE levels were compared between the two time points using a two-tailed Wilcoxon matched-pairs signed-rank test. Lastly, a statistically significant difference was investigated for CPa9-HNE and the commercial calprotectin assay independently using a Kruskal–Wallis test with Dunn’s multiple comparisons test. A receiver-operating characteristics (ROC) curve analysis was used to determine the specificity and sensitivity between all subject groups in the serum for the CPa9-HNE (neoepitope) and the commercial total calprotectin (non-neoepitope) assay.

## 3. Results

### 3.1. Basic Demographics

Basic characteristics are listed in Table 1 for healthy controls, IPF, and COPD participants in cohorts 1 and 2. A significant difference in age was found between the subject groups, with healthy controls being the youngest in both cohorts 1 and 2. This mostly held true for each participant group in cohort 1 when conducting pairwise comparisons of age between healthy and COPD (*p <* 0.0001), healthy and IPF (*p* = 0.0051), but was not observed between the COPD and IPF participant groups (*p* = 0.1). In cohort 2, a statistical difference was found for all participant groups when conducting a pairwise comparison analysis for differences in age (*p* ≤ 004). Overall, sex distribution was also significantly different between the groups, which remained the case in cohort 1 for pairwise comparisons between healthy and COPD (*p* = 0.01), IPF and COPD (*p* = 0.001), with the exception of the IPF and healthy (*p* = 0.1) participant group. When conducting pairwise comparisons of the participant sex distribution in cohort 2, statistical significance was found between IPF and COPD (*p* = 0.005), IPF and healthy (*p* = 0.0009), but not between healthy and COPD (*p* = 0.4). The mean FVC % predicted was similar between COPD and IPF subjects when data were available. As expected, the mean FEV_1_% predicted was significantly lower for COPD compared to IPF subjects when data were available. Most COPD participants had severe disease by the GOLD standards; however, they were considered stable in disease at all visits.

### 3.2. Neutrophilic Activity, Quantified by the Neoepitope-Specific CPa9-HNE Assay, Is Significantly Increased in COPD and IPF Participants

**Figure 1:**
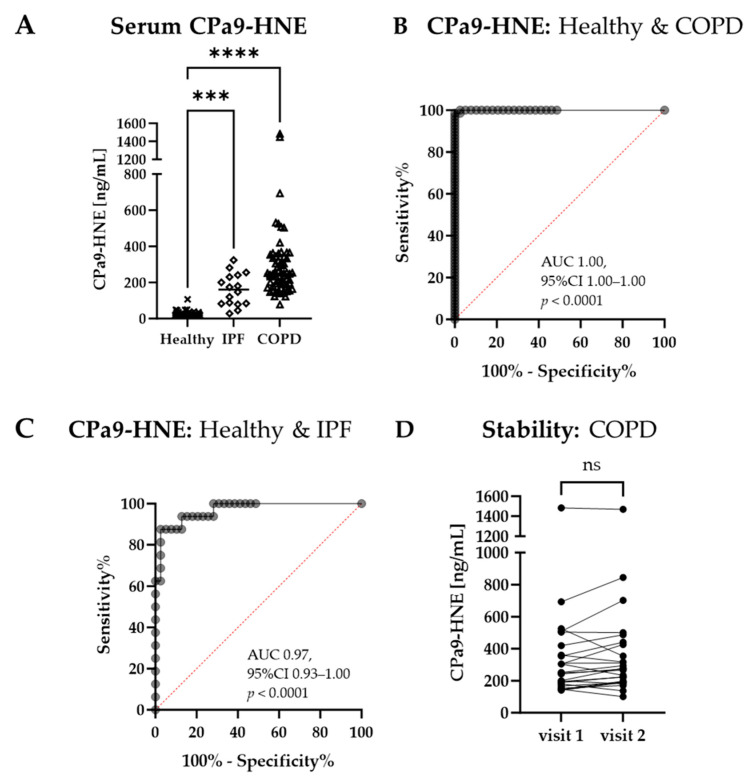
Diagnostic potential of CPa9-HNE in COPD and IPF. (**A**) Serum CPa9-HNE levels in healthy (*n* = 39), COPD (*n* = 67), and IPF (*n* = 16). Data are shown as scatter dot plots with the median indicated with a line and individual participant values of healthy controls indicated with an X shape, IPF indicated with a diamond shape, and COPD indicated with a triangle shape. A statistically significant difference was determined using the Kruskal–Wallis test with Dunn’s multiple comparisons test comparing the COPD and IPF to the healthy subjects. (**B**) ROC analysis of COPD subjects as compared to healthy controls. (**C**) ROC analysis of IPF subjects as compared to healthy controls. (**D**) A parallel dot plot comparing serum CPa9-HNE levels at two visits four weeks apart for 25 clinically stable COPD participants, where data were analyzed using a two-tailed Wilcoxon matched-pairs signed rank. 95% CI, 95% confidence interval; AUC, area under the curve; IPF, idiopathic pulmonary fibrosis; COPD, chronic obstructive pulmonary disease; ns, no significance; and ROC, receiver-operating characteristics. *** *p <* 0.001; and **** *p <* 0.0001.

Serum CPa9-HNE levels for healthy, COPD, and IPF subjects are illustrated in Figure 1A. A significant increase was seen in COPD (baseline median 247.4 [IQR 179.2–334.7] ng/mL, *p <* 0.0001) and IPF (median 161.0 [IQR 82.86–238.5] ng/mL, *p* = 0.0001) as compared to healthy subjects (median 18.94 [IQR 18.32–32.29] ng/mL). When comparing IPF and COPD participants of cohort 1, a trend was observed for the COPD group having a higher biomarker level than the IPF group (*p* = 0.06). A ROC analysis for COPD versus healthy subjects had an area under the curve (AUC) of 1.00 (95% CI 1.00–1.00, *p <* 0.0001; Figure 1B). IPF subjects had an AUC of 0.97 (95% CI 0.93–1.00) when compared to healthy controls (*p <* 0.0001; Figure 1C). To evaluate the stability of CPa9-HNE over time, 25 of the clinically stable COPD subjects were re-evaluated in serum at a four-week visit. CPa9-HNE levels remained stable over the four-week period with no significant change in concentration (follow-up median 273.8 [IQR 190.7–433.3] ng/mL, *p* = 0.0588), as shown in Figure 1D.

The clinical parameters listed in Table 1 for cohort 1 were investigated for associations with CPa9-HNE serum levels. FVC % predicted and CPa9-HNE showed a mild but significant negative correlation in COPD participants (r = −0.2577, *p* = 0.0488); however, not in IPF (r = 0.1859, *p* = 0.491). Healthy females (32.29 [IQR 18.32-47.33] ng/mL) had significantly higher CPa9-HNE levels than healthy males (18.32 [IQR 18.32-23.02] ng/mL; *p* = 0.0468). However, this difference was not observed for COPD participants (*p* = 0.975). No correlation between age and CPa9-HNE was found for any groups (*p* ≥ 0.3). Similarly, no correlation was found between BMI and CPa9-HNE in COPD participants (*p* = 0.8).

### 3.3. The Neoepitope CPa9-HNE Had Superior Diagnostic Power to Identify Disease and Healthy Participants Compared to Non-Neoepitope Measurements in the Serum

**Figure 2:**
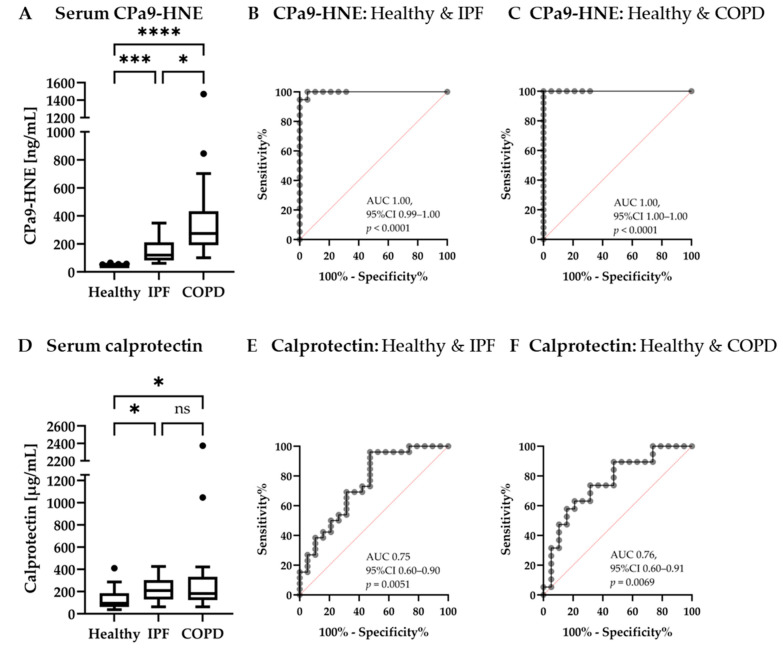
Diagnostic potential of CPa9-HNE (neoepitope) and calprotectin (non-neoepitope) measurements for serum CPa9-HNE (**A**) and serum calprotectin (**D**) levels were compared for 19 healthy controls; 19 participants with an IPF diagnosis, and the 25 COPD participants in a head-to-head comparison, showing data as Tukey’s boxplot with possible outliers indicated as dots ROC analysis was performed for CPa9-HNE in IPF (**B**) and COPD (**C**) when compared to healthy controls, as well as for total calprotectin in IPF (**E**) and COPD (**F**) when compared to healthy controls. 95% CI, 95% confidence interval; AUC, area under the curve; IPF, idiopathic pulmonary fibrosis; COPD, chronic obstructive pulmonary disease; ns, no significance; and ROC, receiver-operating characteristics. * *p <* 0.05; *** *p <* 0.001; and **** *p <* 0.0001.

The calprotectin neoepitope CPa9-HNE was compared head-to-head with a commercial assay for total calprotectin. As seen in Figure 2A, a significant increase in the neoepitope CPa9-HNE for both COPD and IPF participants was again observed when compared to the healthy controls in this cohort: CPa9-HNE levels were significantly elevated in COPD (median 273.8 [IQR 190.7–433.3] ng/mL, *p <* 0.0001) and IPF (120.3 [IQR 81.40–211.0] ng/mL, *p* = 0.0005) as compared to healthy controls (median 44.04 [IQR 44.04–46.52] ng/mL). Moreover, COPD subjects had significantly higher CPa9-HNE levels than IPF (*p* = 0.0111). As seen in Figure 2D, non-neoepitope calprotectin serum levels were also significantly elevated in COPD (median 182.7 [IQR 63.14–332.9] µg/mL, *p* = 0.0219) and IPF (median 208.5 [IQR 128.8–303.4] µg/mL, *p* = 0.0137) when compared to healthy controls (median 94.69 [IQR 61.46–185.3] µg/mL). However, there was no significant difference between COPD and IPF participants for non-neoepitope total calprotectin measurements (*p* > 0.9999). CPa9-HNE biomarker levels had an AUC of 1.00 for both IPF (95% CI 0.99–1.00, *p* < 0.0001) and COPD (95% CI 1.00–1.00, *p* < 0.0001) when compared to healthy controls (Figure 2B,C). Total serum calprotectin levels in IPF and COPD had an AUC of 0.76 (95% CI 0.60–0.90, *p* = 0.0051) and 0.75 (95% CI 0.60–0.91, *p* = 0.0069), respectively, when compared to healthy controls (Figure 2E,F). Additionally, CPa9-HNE biomarker levels had an AUC of 0.68 (95%CI 0.52–0.84), *p* = 0.045) between IPF and COPD subjects, where total calprotectin had an AUC of 0.50 (95%CI 0.33–0.67, *p* = 0.98). Clinical parameters listed in Table 1 for cohort 2 were investigated for associations of CPa9-HNE or non-neoepitope calprotectin levels. For the COPD participants, a negative correlation was found for FVC % predicted and CPa9-HNE (r = −0.458, *p* = 0.0280) but not when measuring calprotectin (r = 0.178, *p* = 0.418). No correlation was found for FEV % predicted for CPa9-HNE (r = 0.249, *p* = 0.251) or intact calprotectin (r = −0.258, *p* = 0.234) measurements. No correlations were found between any of the remaining parameters listed or biomarker measurements.

## 4. Discussion

In the current study, we showed that serum CPa9-HNE was highly upregulated in COPD and IPF compared to healthy controls. Interestingly, the neoepitope-specific marker, CPa9-HNE, separated the disease groups from the healthy group to a larger extent than non-neoepitope measurements for calprotectin in a head-to-head comparison. The serological biomarker CPa9-HNE is associated with the degradation of calprotectin via HNE, which is generated during a NET response from neutrophils [16], thus reflecting the neutrophil activity assessed in stored serum samples. COPD is characterized by chronic inflammation [2,41], which is in line with the current results showing highly elevated serum levels of CPa9-HNE compared to healthy controls. IPF is mainly characterized by fibrosis; however, participants may experience chronic inflammation to some extent [5], which is also in line with the results shown in this study, where CPa9-HNE was significantly increased in IPF compared to healthy controls but to a lesser extent for total calprotectin. CPa9-HNE was able to identify COPD and IPF subjects from healthy controls with high AUCs as computed using ROC analyses. Hence, neutrophil activity may be distinctly different when comparing COPD and IPF to healthy participants. Additionally, CPa9-HNE serum levels in COPD were significantly increased compared to IPF in cohort 2 (Figure 2A) while showing a trend in cohort 1 (Figure 1A), which is in line with COPD having chronic inflammation as a disease hallmark and while fibrotic changes better characterize IPF.

Levels of the serum CPa9-HNE remained stable over a four-week period in COPD participants that were deemed clinically stable, suggesting that CPa9-HNE does not fluctuate and is a stable measure of neutrophil activity over time. Despite the lack of a recent exacerbation event for these COPD participants, a distinct observation was the two outliers with very high serum CPa9-HNE. None of the available basic demographics could explain the very high levels seen in these individuals. However, the participant who participated in the follow-up visit remained clinically stable and at a stable high CPa9-HNE level over time. This suggests that even high levels of neutrophil activity may be found in stable participants, indicating a stable inflammatory state.

It has previously been reported that there is no association between age and BMI with CPa9-HNE serum levels [16], which is in line with observations in this study when data were available. Previously, low BMI has been related to disease severity in COPD in addition to progression, risk of hospitalization, mortality, and tolerability to certain therapies in IPF [42,43]. Thus, a limitation of this study includes the lack of BMI data for the IPF and healthy participant group, even though current data do not indicate this. In the healthy group, a difference was observed between sexes within cohort 1, but this was not the case in any other group in this study. It was not possible to establish if there was a sex-related difference in the IPF group due to the low number of females present in both cohorts. Thus, a limitation of this study was the participants not being matched in age or sex, which could potentially have an effect on their CPa9-HNE levels, although the data of this study or others do not suggest this. In previous studies, serum CPa9-HNE levels for healthy controls have been reported at a median of 42.7 ng/mL [16], which is in line with the second healthy control group (*n* = 19, median 44.04 ng/mL) but different from the control group in cohort 1 (*n* = 39, median 18.94 ng/mL). Limited information was available for the healthy control groups for both cohorts, where future studies with more comprehensive information might offer a more precise characterization of CPa9-HNE in healthy subjects.

In general, CPa9-HNE was not associated with spirometry measures, albeit a negative correlation between FVC and CPa9-HNE was shown in the COPD participants. No correlation was observed for FVC and FEV_1_ with IPF and COPD, respectively. However, it is more appropriate to consider CPa9-HNE as a measure of disease activity rather than severity when considering that it is generated during NETosis from activated neutrophils as part of inflammation [16]. Therefore, we theorize that CPa9-HNE might be seen in flares during periods of high disease activity when observed over time for the participant, whereas levels would be expected to be lower in periods of inactive disease. This is supported by previous observations in inflammatory bowel diseases, where CPa9-HNE serum levels correlated with endoscopic disease activity for ulcerative colitis and Crohn’s disease [16]. However, future studies are needed to fully elaborate on the role of CPa9-HNE related to disease activity and/or the severity in COPD and IPF with larger and well-described cohorts. Since CPa9-HNE reflects neutrophil activity, it might potentially predict and characterize inflammatory events more easily than current tools. Currently, the rapid decay of neutrophils in blood samples usually demands neutrophil activity assessment quickly after blood collection [11,44], where neutrophilic count does not reflect disease activity. In contrast, the stability of CPa9-HNE neoepitope storage has been shown in serum samples by testing neoepitope recovery over at least four freeze/thaw cycles and storage at room temperature for >24 h and longer at 4 °C [16].

The neoepitope-specific CPa9-HNE biomarker was compared to the commercial MRP8/14 assay, measuring total non-neoepitope calprotectin in a direct comparison. Significantly elevated levels of both CPa9-HNE and total calprotectin were found for both COPD and IPF compared to healthy controls. However, CPa9-HNE had the best ability to distinguish the disease groups from the healthy reference group, where the AUC for CPa9-HNE in IPF and COPD was equal to 1.00, and the AUC for non-neoepitope calprotectin measurements in IPF and COPD were 0.75 and 0.76, respectively, thus showing a relevance for neoepitope measurements in disease characterization. Moreover, it was also possible to distinguish IPF from COPD participants (Figure 2A) using CPa9-HNE, which was not possible with measurements of total calprotectin (Figure 2D). These results highlight the difference between measuring a specific cleavage site mediated by protease activity, a neoepitope, in calprotectin versus measuring total calprotectin since the neoepitope signifies both release of calprotectin but also its degradation with HNE at the specific cleavage site [16,45]. Moreover, it has also been demonstrated that different neoepitopes, despite their same protein origin, have different meanings, e.g., some being indicators of protein production while others proteolytic degradation [46,47,48], highlighting that the specific epitope is important [49].

Limitations to the current study include the lack of data availability on smoking history for the IPF and healthy participant groups, where it would be interesting to incorporate smoking history for all participant groups to investigate and compare biomarker levels between all participant groups. Future studies should aim to include this information. Additionally, the subject groups investigated in this study had a lower number of participants, non-matched in clinical parameters; therefore, this study served as a proof-of-concept study that should be expanded upon in the future.

## 5. Conclusions

We have demonstrated in this study that calprotectin degradation via HNE is significantly upregulated in COPD and IPF, signified by the serological biomarker CPa9-HNE that quantifies a neoepitope of a calprotectin degradation fragment that is associated with neutrophil activity. The results of this study support that neutrophil activity is an essential common denominator for both disease indications, which can be easily assessed even in stored serum samples via CPa9-HNE. COPD subjects had the most pronounced increase in CPa9-HNE, which is in line with it being a more inflammatory-driven disease. Moreover, CPa9-HNE was better at distinguishing the groups of healthy, COPD, and IPF than total calprotectin serum measurements, highlighting the importance of assessing neutrophil activity in these participants and the benefit of neoepitope measurements in connection with lung diseases. Future studies are needed to elucidate the role of neutrophil activity and the full potential of CPa9-HNE as a neoepitope-specific biomarker for the lung diseases investigated here and other diseases with an inflammatory component.

## Figures and Tables

**Table 1:** Basic demographics for healthy, IPF, and COPD participants for the two cohorts.

**Cohort 1: Demographics of Participants for the CPa9-HNE Evaluation **				
	**Healthy**	**COPD**	**IPF**	***p*-Value**
*n*	39	67	16	-
Age (yrs), mean ± SD	45 ± 16	71 ± 9	65 ± 7	*p <* 0.0001 #
Male/female sex, *n* (%)	26/13 (66.7%/33.3%)	28/39 (41.8%/58.2%)	14/2 (87.5%/12.5%)	*p* = 0.0011 £
BMI (kg/m^2^), mean ± SD	-	24.5 ± 6.2	-	-
Current/ex/never smoker, *n*	-	11/53/3 (16.4%/79.1%/4.5%)	-	-
GOLD A/B/C/D, *n*	-	4/13/6/44 (6%/19.4%/8.9%/65.7%)	-	-
FVC (% pred), mean ± SD	-	66.1 ± 17.3	74.5 ±12.8	*p* = 0.0735 $
FEV_1_ (% pred), mean ± SD	-	39.9 ± 15.8	79.0 ± 8.9	*p <* 0.0001 $
**Cohort 2: Demographics of Participants for the Head-to-Head Comparison of CPa9-HNE and Calprotectin**				
	**Healthy**	**COPD** **Visit 2**	**IPF**	***p*-Value**
*n*	19	25	19	-
Age (yrs), mean ± SD	36.8 ± 10.11	72.7 ± 8.2	63.9 ± 7.7	*p <* 0.0001 #
Male/female sex, *n* (%)	5/12 (29.4%/70.6%)	11/15 (42.3%/57.7%)	16/3 (84.2%/15.8%)	*p* = 0.0021 £
BMI (kg/m^2^), mean ± SD	-	24.1 ± 4.9	-	-
Current/ex/never smoker, *n*	-	1/22/2 (4%/88%/8%)	-	-
GOLD A/B/C/D, *n* (%)	-	0/0/1/24 (0%/0%/4%/96%)	-	-
FVC (% pred), mean ± SD	-	45.3 ± 21.9	-	-
FEV_1_ (% pred), mean ± SD	-	48.4 ± 15.6	-	-

BMI, body mass index; FEV_1_, forced expiratory volume in 1 s; FVC, forced vital capacity; GOLD, Global Initiative for Chronic Obstructive Lung Disease; COPD, chronic obstructive pulmonary disease; IPF, idiopathic pulmonary fibrosis; pred, predicted; yrs, years; and SD, standard deviation. # Kruskal–Wallis test across all groups, £ χ^2^-test, $ Mann–Whitney test.

## Data Availability

The datasets and/or data analysis generated during the study are not publicly available due to restrictions of Danish data protection laws; however, they are available from the corresponding author upon reasonable request.

## Abbreviations

The area under the curve (AUC), body-mass index (BMI), chronic obstructive pulmonary disease (COPD), specific calprotectin protein fragment generated by human neutrophil elastase (CPa9-HNE), enzyme-linked immunosorbent assay (ELISA), forced expiratory volume during the first second (FEV_1_), forced vital capacity (FVC), Global Initiative for Obstructive Lung Disease (GOLD), human neutrophil elastase (HNE), idiopathic pulmonary fibrosis (IPF), neutrophil extracellular traps (NETs), receiver operating characteristics (ROC), standard deviation (SD), and years (yrs).
